# Computer aided quantification for retinal lesions in patients with moderate and severe non-proliferative diabetic retinopathy: a retrospective cohort study

**DOI:** 10.1186/1471-2415-14-126

**Published:** 2014-10-31

**Authors:** Huiqun Wu, Xiaofeng Zhang, Xingyun Geng, Jiancheng Dong, Guomin Zhou

**Affiliations:** Department of Medical Informatics, Medical School of Nantong University, Nantong, 226001 China; Key Laboratory of Medical Imaging Computing and Computer Assisted Intervention (MICCAI) of Shanghai, Shanghai, 200032 China; School of Computer Science and Technology, Nantong University, Nantong, 226001 China; Department of Anatomy, Histology and Embryology, Shanghai Medical College of Fudan University, Shanghai, 200032 China

**Keywords:** Diabetic retinopathy, Fundus lesions, Exudation, Microaneurysms

## Abstract

**Background:**

Detection of retinal lesions like micro-aneurysms and exudates are important for the clinical diagnosis of diabetes retinopathy. The traditional subjective judgments by clinicians are dependent on their experience and can be subject to lack of consistency and therefore a quantification method is worthwhile.

**Methods:**

In this study, 10 moderate non-proliferative diabetes retinopathy (NPDR) patients and 10 severe NPDR ones were retrospectively selected as a cohort. Mathematical morphological methods were used for automatic segmentation of lesions. For exudates detection, images were pre-processed with adaptive histogram equalization to enhance contrast, then binary images for area calculation were obtained by threshold classification. For micro-aneurysms detection, the images were pre-processed by top-hat and bottom-hat transformation, then Otsu method and Hough transform were used to classify micro-aneurysms. Post-processing morphological methods were used to preclude the false positive noise.

**Results:**

After segmentation, the area of exuduates divided by optic disk area (exudates/disk ratio) and counts of microaneurysms were quantified and compared between the moderate and severe non-proliferative diabetic retinopathy groups, which had significant difference(P < 0.05).

**Conclusions:**

In conclusion, morphological features of lesion might be an image marker for NPDR grading and computer aided quantification of retinal lesion could be a practical way for clinicians to better investigates diabetic retinopathy.

## Background

Early detection of eye disease due to diabetes, glaucoma, and age-related macular degeneration has a significant impact on the prevention of blindness. It’s estimated that nearly one million patients would be screened every day worldwide for diabetic retinopathy(DR) by 2025. Detecting and counting lesions in the human retina like microaneurysms and exudates is important for clinical diagnosis of DR [1, 2], but is also a time-consuming task for ophthalmologists and open to human error. That is why much effort has been done to detect lesions in the human retina automatically. In this study, we proposed a computerized framework for automatic detection of exudate and microaneurysms and compared the morphological features in moderate and severe non-proliferative diabetic retinopathy.

## Methods

### Dataset selection and preparation

During November 2012 to October 2013, 20 patients diagnosed with DR by fundus image but without cataract degeneration, optic disk edema, macular degeneration, retinal vessel obstruction which could affect retinal images were included in our study. For these patients, 10 patients (7 males and 3 females, mean age: 60.8 ± 11.0 years old) were graded as moderate non-proliferative diabetes retinopathy (NPDR), and another 10 ones (6 males and 4 females, mean age: 63.2 ± 12.1) were graded as severe NPDR according to the international classification for NPDR [3]. All the patients had been diagnosed with diabetes, with the disease course ranged from 8 to 16 years, mean age: 9.4 ± 4.9 years old. All the fundus images were obtained with the same 45° field of view (FOV) camera, with the macula at the center. The image acquisition conditions were consistent at 3504 × 2336 pixels. The patients accompanied with hypertension were excluded. The study protocol was conducted in accordance with the ethical guidelines of the 1995 Declaration of Helsinki. This study was approved by Ethics Committee of Nantong University. Informed consent was obtained from all patients. Before next processing, the size of tested images was reduced in size to 685 × 584 pixels and calibrated to prove the measurement of pathological changes comparable.

### Exudate detection

**Figure 1:**
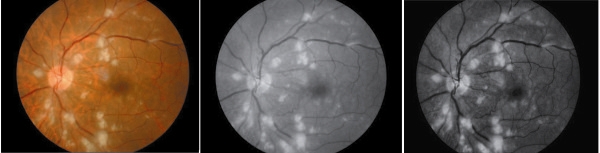
**The pre-processing of original retinal image (left) in green channel (middle) and using adaptive histogram equation algorithm (right).**

The original retinal images were pre-processed, and the green channel of RGB image was obtained to enhance the contrast between background and foreground tissues. In this study, adaptive histogram equalization algorithm was firstly used to improve the image intensity contrast.


in which: *J*_*n*_ is the gray values after treatment; *I*_max_, *I*_min_ is the largest and lowest gray values in original image respectively; *I*_*n*_ is the gray value before treatment; *a* is luminance compensation coefficient. After processing, the exudates had higher contrast which was easier for further segmentation (Figure 1).

For exudates detection, different approaches have been proposed. We firstly adopted traditional threshold classification protocol to coarsely classify the background and exudate areas. Then, morphological operators including erosion and dilation were performed on segmented binary image to exclude the noise. To prevent the influence of pixel calibration, the area of total exudates were divided by the area of optic disk and this value was called exudates/disk ratio.

### Microaneurysm detection

**Figure 2:**
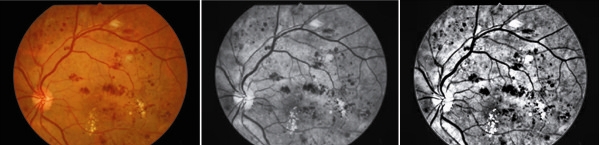
**Top-bottom hat transform.**

The microaneurysm has its own morphological features that could be used to distinguish from hemorrhage. It is usually presented as red dots, with 12-100 μm diameters, and could be distinguished in fundus images if the diameter of which was over 30 μm. The pre-processing steps for microaneurysm was similar as above described. First, green channel of RGB image was extracted. Then adaptive histogram equalization was used to enhance contrast after the image was transformed to gray image. Further, top-bottom hat transform was implemented to enhance the microaneurysm intensity (Figure 2).

in which *b* is a structure element.

Due to the confused intensity contrast among aneurysm, hemorrhage and blood vessels, herein, we proposed two computerized framework to detect aneurysms. First, we adopted Otsu algorithm which is a statistical method that calculates the probability of each pixel as *C*_0_ and *C*_1_ by threshold. Then, the best threshold for intra-class variance was calculated as:


in which , , , *n*_*i*_ denotes the number of pixels with gray value i. Then, morphological post-processing steps were performed on the binary logic image to detect microaneurysms.

The other protocol used in this study for microaneurysm detection is based on Hough transform. Hough detection is a useful method to detect line and circular features in images, which transforming the pixels in original image to parameter coordinate [4]. In this way, linear arranged pixels with the same slope and intercept are shown as the same pixel in the parameter coordinates. Compared to a line, a circle is actually simpler to represent in parameter space, since the parameters of the circle can be directly transformed into the parameter space as x = a + rcos(θ), y = b + rsin(θ). And the location of circle center from the accumulator data could be determined in the parameter space represents.

### Statistical analysis

In this experiment, the exudates/disk ratio and number of microaneurysms were expressed as *x*^-^ ± SD. Student T test was used to compare the area of exudates and number of microaneurysms. The P value less than 0.05 was treated as statistically significant.

## Results

**Figure 3:**
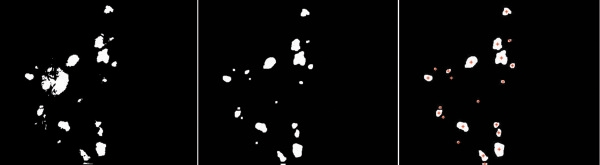
**Exudates detection and labeling.**

After threshold segmentation and labeling, the numbers and areas of each exudate were automatically obtained for further analysis (Figure 3), after statistical analysis, the exudates/disk ratio in the severe group (3.2 ± 1.1) increased than that of the moderate group (1.8 ± 1.1) (P < 0.05).

**Figure 4:**
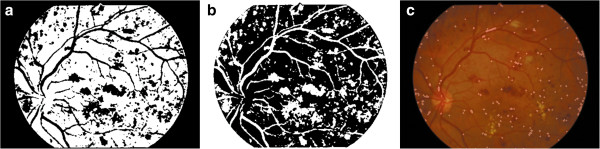
**The segmentation of microaneurysms by morphological treatments.** (4**a**: left panel; 4**b**: middle panel; 4**c**: right panel).

After Otsu segmentation (Figure 4a), the binary values were inverted to make blood vessels and microaneurysms as white structures (Figure 4b). The original image was subtracted by the image after erosion (2-pixel disk structure element) and further dilation (1-pixel disk structure element) was performed to obtain microaneurysms (Figure 4c).

**Figure 5:**
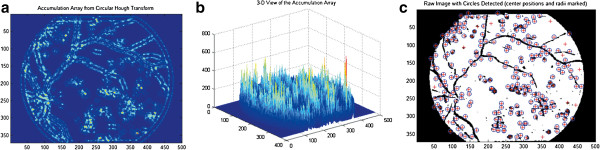
**Hough detection results in 2D, 3D coordinates and overlapped in segmented image.** (5**a**: left panel; 5**b**: middle panel; 5**c**: right panel).

For Hough detection, the highest peaks are corresponded to a particular radius in the accumulator data (Figure 5a). If the height of the peaks is equal compared to the number of edge pixels for a circle with the particular radius, the coordinates of the peaks does probably correspond to the center of such a circle (Figure 5b). In this study, we identified the number of microaneurysms automatically and overlapped the results in segmented retinal images (Figure 5c).

In this study, the number of microaneurysms in the moderate group were 14.5 ± 2.3; while was 27.5 ± 5.7 in the severe group, with significant difference (P < 0.05).

## Discussion

In clinical practices, fundus image observation could help clinicians to detect the microcirculation changes in vivo. However, such observation was usually dependant on the observers’ experience. Although some guidelines for qualification of lesions in four quadrants of retinal image, the description of different amounts of aneurysms and exudates increases clinicians’ work loads. Nowadays, different standards for DR have been published [5], morphological features of lesions are commonly mentioned parameters for disease severity grading. For any automatic detection and screening system developed for retinal illnesses, the detection of morphological structures such as the optic disc, macula, and vessels is extremely important. For retinal image analysis, a robust automatic algorithm could be applied to quantify the retinal vessel width, exudates, hemorrhage, microaneurysms, relieving the work load by ophthalmologists. Besides, such quantification data could be stored and distributed easily, thus significant for diagnosis and prognosis research compared with the simply observational experience made by clinicians. Some systems have been developed to determine DR stages—normal, mild moderate NPDR, severe NPDR and PDR stages [6–9]. In our further study, we plan to implement blood vessel geometric features for comparative analysis.

The computer framework used in our study was mainly based on mathematical morphological analysis, from segmentation and measurement, which is different from machine learning methods that train the classifier based on textual or intensity features and output the classified result as grades [10–14]. The latter way is a system-oriented framework that could determine the result, but lack of number values data output. Such number values are essential in clinical investigation as an image marker for diagnosis and prognosis. Meanwhile, such un-supervised segmentation technique could work without training samples and therefore could be applied into fundus image directly, which provides convenience for clinical practices. Besides applications in DR, morphologic structures, retinal parameters, and changes in retinal features are utilized in the automatic detection of retinal pathologies such as age-related macular degeneration (ARMD), glaucoma, and optic nerve hypoplasia (ONH) [15–17].

There are also some weaknesses for our computer framework in clinical practice. Taking microaneurysms’ detection for example, if no apriori knowledge is known about the size of microaneurysms then this process could be quite challenging. And the center of a circle can also be represented by a peak with a height less than the number of edge pixels, the incomplete or ellipse shaped circle will increase the segmented errors. Another limitation in our study is that the variation of FOV from different digital fundus cameras could affect the exudates/disk ratio because of different observation areas are investigated.

## Conclusions

In conclusion, a computer framework based on mathematical morphological analysis could be utilized as a reference for clinicians to quantify exudates and microaneurysms in NPDR, and larger exudate area and more aneurysm counts might help grading the NPDR, larger samples and prospective clinical trials are needed for further validation.

## References

[1] Klein R, Meuer SM, Moss SE, Klein BE (1995). Retinal aneurysm counts and 10-year progression of diabetic retinopathy. Arch Ophthalmo.

[2] Kohner EM, Stratton IM, Aldington SJ, Turner RC, Matthews DR (1999). Microaneurysms in the development of diabetic retinopathy (UKPDS 42). Diabetologia.

[3] RCO (2005). Guidelines for Diabetic Retinopathy 2005.

[4] Mohamed R, Haniza Y, Puteh S, Ali YMS, Abdul RS, Masanori S, Sazali Y, Mamat MR, Karthigayan M (2005). Object detection using circular hough transform. Am J Appl Sci.

[5] Chen Z, Zhang SS, Zhu HM (2011). Analysis of international clinical diabetic retinopathy disease severity scale. Int J Opthalmol.

[6] Sopharak A, Uyyanonvara B, Barman S, Williamson TH (2008). Automatic detection of diabetic retinopathy exudates from non-dilated retinal images using mathematical morphology methods. Comput Med Imaging Graph.

[7] Walter T, Klein JC, Massin P, Erginay A (2002). A contribution of image processing to the diagnosis of diabetic retinopathy-detection of exudates in color fundus images of the human retina. IEEE Trans Med Imaging.

[8] Yun WL, Rajendra Acharya U, Venkatesh YV, Cheec C, Minb LC, Ng EYK (2008). Identification of different stages of diabetic retinopathy using retinal optical images. Inf Sci.

[9] Nayak J, Bhat P, Acharya UR, Lim CM, Kagathi M (2008). Automated identification of diabetic retinopathy stages using digital fundus images. J Med Syst.

[10] Narasimha-Iyer H, Can A, Roysam B, Stewart V, Tanenbaum HL, Majerovics A, Singh H (2006). Robust detection and classification of longitudinal changes in color retinal fundus images for monitoring diabetic retinopathy. IEEE Trans Biomed Eng.

[11] Stewart CV (2005). Computer vision algorithms for retinal image analysis: current results and future directions. Lect Notes Comput Sci.

[12] Narasimha-Iyer H, Beach JM, Khoobehi B, Roysam B (2007). Automatic identification of retinal arteries and veins from dual-wavelength images using structural and functional features. IEEE Trans Biomed Eng.

[13] Gagnon L, Lalonde M, Beaulieu M, Boucher MC (2001). Procedure to detect anatomical structures in optical fundus images. Proc Conf on Medical Imaging.

[14] Kara S, Güven A, Öztürk Öner A (2006). Utilization of artificial neural networks in the diagnosis of optic nerve diseases. Comput Biol Med.

[15] Niemeijer M, Abramoff MD, van Ginneken B (2007). Segmentation of the optic disc, macula and vascular arch in fundus photographs. IEEE Trans Med Imag.

[16] Foroozan R (2005). Superior segmental optic nerve hypoplasia and diabetes mellitus. J Diabetes Complicat.

[17] Riverón EMF, del Toro-Céspedes M (2004). Measurement of parameters of the optic disc in ophthalmoscopic color images of human retina. Lect Notes Comput Sci.

[CR18] The pre-publication history for this paper can be accessed here: http://www.biomedcentral.com/1471-2415/14/126/prepub

